# Ring Vaccination and Smallpox Control

**DOI:** 10.3201/eid1005.030419

**Published:** 2004-05

**Authors:** Mirjam Kretzschmar, Susan van den Hof, Jacco Wallinga, Jan van Wijngaarden

**Affiliations:** * National Institute of Public Health and the Environment (RIVM), Bilthoven, the Netherlands; † Inspectorate of Health Care, the Hague, the Netherlands

**Keywords:** smallpox, ring vaccination, mathematical model

## Abstract

We present a stochastic model for the spread of smallpox after a small number of index cases are introduced into a susceptible population. The model describes a branching process for the spread of the infection and the effects of intervention measures. We discuss scenarios in which ring vaccination of direct contacts of infected persons is sufficient to contain an epidemic. Ring vaccination can be successful if infectious cases are rapidly diagnosed. However, because of the inherent stochastic nature of epidemic outbreaks, both the size and duration of contained outbreaks are highly variable. Intervention requirements depend on the basic reproduction number *R_0_*, for which different estimates exist. When faced with the decision of whether to rely on ring vaccination, the public health community should be aware that an epidemic might take time to subside even for an eventually successful intervention strategy.

Recently, concerns about a bioterror attack with the smallpox virus or other infectious disease agents have risen (*1**,**2*). While new vaccines with fewer adverse consequences are being developed (*3*), the existing vaccines, which have potential side effects and a not negligible lethality, are the only vaccines available (*4*,*5*). In the United States during the recent voluntary smallpox vaccination program, a limited number of healthcare workers volunteered for vaccination because of the low risks associated with vaccination and the low risk for infection (*6*). If an outbreak occurs, vaccination strategies include ring vaccination around diagnosed cases of smallpox or a mass vaccination to begin as soon as the first cases are diagnosed. Without natural smallpox infections, practical experience with ring vaccination against smallpox cannot be gained; accounts of the vaccination programs that eradicated smallpox in the 1970s are the only source of information (*7*). Combined with information collected during the last decades of smallpox circulation, mathematical modeling offers a tool to explore various vaccination scenarios if an outbreak occurs (*8**–**15*).

We investigated which conditions are the best for effective use of ring vaccination, a strategy in which direct contacts of diagnosed cases are identified and vaccinated. We also investigated whether monitoring contacts contributes to the success of ring vaccination. We used a stochastic model that distinguished between close and casual contacts to explore the variability in the number of infected persons during an outbreak, and the time until the outbreak is over. We derived expressions for the basic reproduction number (*R_0_*) and the effective reproduction number (*R_υ_*). We investigated how effectiveness of ring vaccination depends on the time until diagnosis of a symptomatic case, the time to identify and vaccinate contacts in the close contact and casual contact ring, and the vaccination coverage required to contain an epidemic.

## Methods

The model describes the number of infected persons after one or more index cases are introduced. It simulates a stochastic process in which every infected person generates a number of new infections according to a given probability distribution. This process implies that contacts of different infected persons are independent of each other and that no saturation of the incidence occurs at higher prevalence. The model is applicable for the first few generations of infection, if the outbreak goes unchecked, and for the complete outbreak if it is contained. We summarize the main features of the model; the formal model definition is given in the Appendix.

### Course of Infection and Transmission

**Figure 1:**
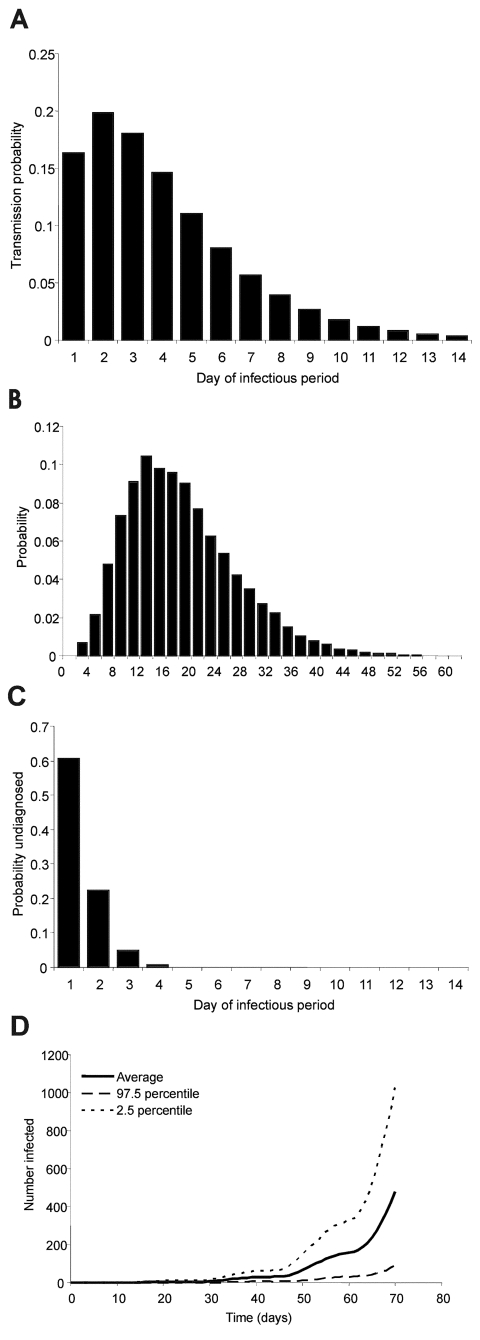
A, the transmission probability per contact by day of the infectious period; B, the probability distribution of the number of contacts with susceptible persons per day; C, the probability of remaining undiagnosed but infectious case by day of the infectious period; and D, the mean (solid line) and the 2.5% and 97.5% percentiles (dotted lines) of the number of infected persons for 500 simulation runs for an epidemic without any intervention after the introduction of one index case at the beginning of this incubation period at t=0.

The noninfectious state (incubation period plus prodromal phase) lasts 12–15 days (*7*,*16*,*17*) with specified probabilities per day of moving to the infectious state. The assumption that infectivity during the prodromal phase is negligible is supported by a recently published statistical analysis of outbreak data (*16*). The duration of the infectious state *D_I_* is 14 days (*7*,*16*,*18*), with variable infectiousness during that time (*13*,*19*,*8*). The probability of transmission per contact p_τ_ , where τ denotes the day of the infectious period, is high at the beginning and low at the end of the infectious period (Figure 1a). At the end of the infectious period, a person either recovers or dies. The case-fatality rate is 30% (*14*), which is an average value for the case-fatality rate of variola major.

Transmission takes place in two rings of contacts: 1) household and other close contacts, and 2) more casual face-to-face contacts. We assumed that in the close contact ring the probability of transmission is five times higher than in the casual contact ring (g = 0.2). The number of contacts on day τ of the infectious period in the close contact ring follows a Poisson distribution with mean μ_τ_^(1)^, and in the casual contact ring this number follows a a negative binomial distribution with mean μ_τ_^(2)^. The values (μ_τ_^(1)^ = 2 and μ_τ_^(2)^ = 14.9) were chosen such that the total number of contacts per day was comparable to numbers observed in empirical studies (*20*) (Figure 1b). For every contact, the event of transmission is determined by the infectiousness by day of the infectious period.

Most people can be infected again, and smallpox can develop 10–20 years after vaccination (*21**,**22*). While residual immunity might lower the case-fatality rate, it might also lead to a later diagnosis for infected persons because disease symptoms are milder. An infection with milder symptoms is probably less infectious, but an infectious person might have more contacts with others because he or she feels less impaired by disease symptoms. Hence, the net effects of residual immunity are difficult to assess. We assumed that all persons are equally susceptible, and that no protective immunity remains in the population from previous vaccination.

The basic reproduction number *R_0_* describes the average number of secondary cases produced from contact with an infected person during the infectious period and without intervention. The number can be computed as the sum of the reproduction numbers in the close contact and casual contact ring


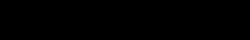


For the baseline parameter values given in the Table, *R_0_* = 5.23, which when broken down by rings of contacts gives 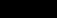 and 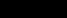, i.e., 40.2% of all transmissions take place in the close contact ring. We use the parameter *a_1_* in the function describing the transmission probability (Table) to vary the basic reproduction number, i.e., if we want to simulate an outbreak under the assumption that *R_0_* is say 5, we chose *a_1_* accordingly. In the literature, the estimates given for *R_0_* vary between 3–6 (*9**,**10**,**23*) and 10–20 (*1*,*14*).

**Table:** The baseline parameter values

Model parameter	Notation	Baseline value
Course of the infection		
Maximum duration latent period	*D_E_*	15 d
Probability of transition to infectious state on day τ of the latent period	*γ_τ_ , τ=1,...,D_E_*	0.0 for τ = 1,...,12 0.3 for τ = 13 0.6 for τ = 14 1.0 for τ = 15
Duration infectious period	*D_I_*	14 days
Case-fatality rate	*f*	0.3
Transmission probability per contact	*p_τ_*	with *a_1_ = 0.27, a_2_ = 0.5*
Ratio of infectiousness of casual contact and close contact ring	*g*	0.2
Contacts		
Mean number of contacts in close contact ring (Poisson distribution)	*μ_τ_^(1)^*	2
Mean number of contacts in the casual contact ring (negative binomial distribution)	*μ_τ_^(2)^*	14.9 (sd 8.4) NegBin(4, 0.212)
Intervention		
Probability of diagnosis	*δ_τ_*	with *b_i_* = 0.5, *b_2_* = 0 (*b_2_*=6 for first index case)
Time needed to trace contact	*r^(i)^*	1 day for i = 1, 3 days for i = 2 (3 days for both rings for first index case)
Time window during which vaccination is effective	*w*	4 days
Vaccination coverage	*c^(i)^*	0.95 for i = 1, 0.5 for i = 2 (0.5 for both rings for first index case)

### Ring Vaccination

**Figure 2:**
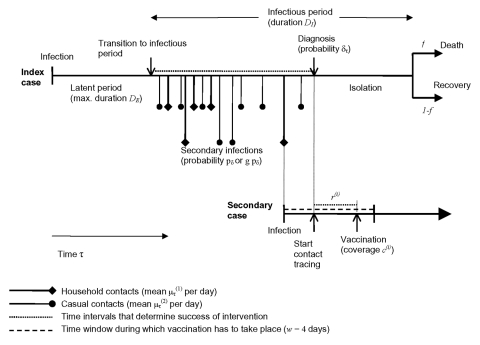
The time course of events in the process of transmission and intervention. The success of intervention is essentially determined by the time between start of the infectious period and diagnosis of the index case, and the time between the start of contact tracing and the vaccination of the contact.

Ring vaccination in the model includes complete isolation of diagnosed symptomatic patients with cases of smallpox and vaccination of (a fraction of) all contacts of the diagnosed patient. In our baseline scenario, we assumed that vaccinated contacts are not isolated after vaccination and may therefore transmit the infection to others if they become infectious. In addition, we enhance the baseline intervention by including monitoring of identified contacts. The effectiveness of the intervention therefore is determined by the probability of diagnosis per day of the infectious period, the time needed to identify contacts of the close contact and casual contact ring, the vaccination coverage in the close contact and the casual contact ring, and whether monitoring of contacts is performed. Some of those parameters (speed of diagnosis and time to identifying contacts) differ between the first index case in the population and cases occurring later in the epidemic. In Figure 2, the timing of the key events in the chain of transmission and intervention is shown schematically. The index patient can cause new cases of infection between the beginning of the infectious period until diagnosis and isolation. For a secondary case, vaccination has to take place within 4 days after infection (*14*) to prevent disease.

We denote with δ_τ_ the probability of diagnosis on day τ of the infectious period for those persons who have not been diagnosed before. From those probabilities, one can derive the probability that an infectious person is not yet diagnosed on day τ of his or her infectious period (Figure 1c). By υ_τ_^(i)^, we denote the probability that a contact in ring i (i = 1 or 2), who was infected on day τ of the index patient’s infectious period, will be vaccinated within 4 days of being infected. In the Appendix, υ_τ_ depends on the diagnosis probabilities, the time needed for contact tracing, and the vaccination coverage. Throughout, we assume that the vaccine efficacy is 100%. We can now determine an effective reproduction number *R_υ_* that describes the number of secondary cases caused by an index patient in a situation with intervention:


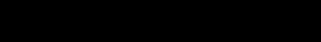


A special strategy included in this formula is an intervention where only case isolation is performed without vaccination of contacts. This formula also applies to an intervention where only case isolation is performed without vaccination of contacts, if the vaccination coverage *c^(i)^* is set to zero. Equivalently, it describes the situation that no window period exists (*24*). The reproduction number can then be calculated as


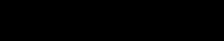


If vaccination is ineffective, but there is monitoring of contacts, the monitoring will have the same effect on *R_0_* as an effective vaccination, because the contacts will not be able to disseminate the virus any further (assuming a fully effective monitoring). Therefore, *R_0_* can be computed with the formula including vaccination, where the window period is now set to w = 15, of the full duration of the infectious period. This assumption means that regardless of when the index patient is diagnosed, contacts can effectively be excluded from further transmission. If monitoring of contacts is not 100% effective, the parameter *c^(i)^* for the coverage can be used to express the extent of successful monitoring.

The outbreak can be controlled if *R_υ_* < 1. In the Table, the model parameters and their baseline values are listed. In the Appendix, the formal model definition is given.

## Results

### Baseline Parameter Set

An epidemic starting with one index case in a completely susceptible population without intervention grows exponentially, if it survives early extinction (minor epidemics). The large range of possible courses of the epidemic reflects the stochastic variability (Figure 1d). If the intervention does not succeed in reducing the effective reproduction number *R_υ_* to below 1, the epidemic will continue to grow exponentially, albeit at a lower rate. For example, if diagnosed infectious persons are isolated, but no ring vaccination is performed, the effective reproduction number is *R_υ_*=1.65 and the epidemic cannot be contained.

**Figure 3:**
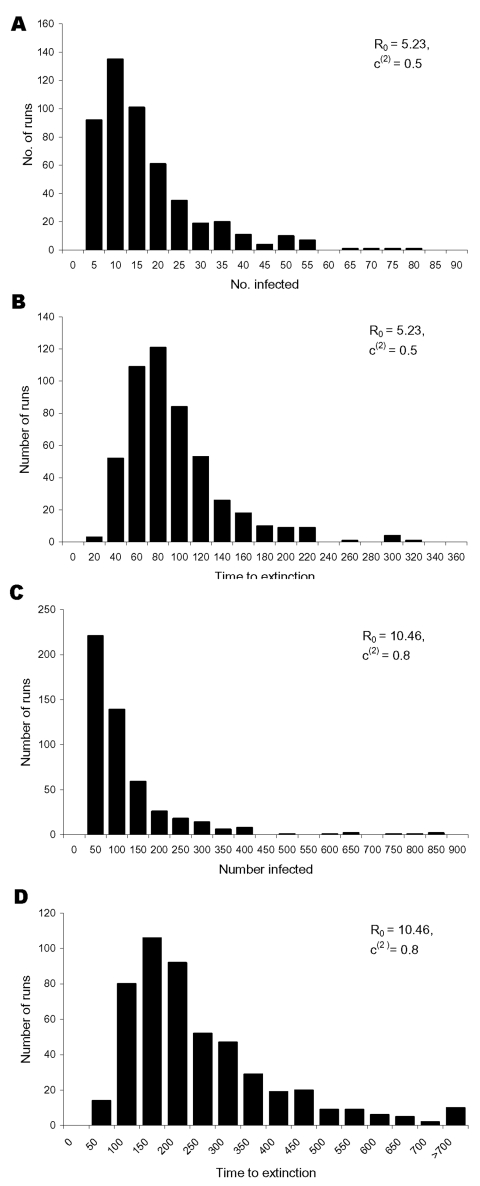
The distribution of A, the total number of infected persons excluding those infected contacts who were vaccinated on time to prevent disease, and B, the time to extinction for 500 simulation runs with the baseline intervention parameter values and a basic reproduction number of 5.23. For a basic reproduction number of 10.46 and an increase of the vaccination coverage in the casual contact ring to 80% in C, the distribution of the total number of infected persons, and in D, the distribution of the time to extinction, is shown for 500 simulation runs.

If the intervention succeeds in reducing the effective reproduction number *R_υ_* to <1, the size of successive generations of infected persons declines. For the parameter values of the baseline scenario given in the Table, we have *R_υ_* = 0.67 and 94.4% of all transmissions take place in the casual contact ring. The epidemic can then be contained and the virus eradicated. In Figure 3a and b, the distribution of the total number of infected persons (excluding those who were vaccinated in time to prevent symptomatic infection) and of the time until recovery of the last infected patient is shown for 500 simulation runs with the baseline parameters (Table). The time until the epidemic is over is quite variable: on average it takes 82 days, in some cases it takes up to 1 year (range 22–334 days). During this time, an average of 209 contacts are vaccinated (range 6–1,038 contacts), with a mean number of 13.5 vaccinated contacts per infected case. On average, 15 persons are infected (range 1–123 persons), if we exclude those infected persons who were vaccinated on time. If we include the infected contacts who were vaccinated on time, the mean number of persons infected is 29 (range 1–200 contacts). On average, 5 persons die of smallpox (range 0–30 persons). If monitoring of contacts is added to the intervention (incorporated in the model by assuming that *w* = 15), the effective reproduction number can be further reduced to 0.53. The average number of infected persons drops to 6 (range 1–43 persons), excluding the identified infected contacts who are vaccinated or monitored and to 14 (range 1–96 persons), including the vaccinated and monitored infected contacts. The mean time to extinction is now 53 days (range 20–288 days). The fraction of transmissions taking place in the casual contact ring is slightly lower. at 93.7%.

To contrast the baseline scenario, in Figure 3c and d we show results for the case that R_0_ = 10.46, i.e., twice the value of baseline scenario. To contain the epidemic, we now assumed that 80% of all contacts in the casual contact ring were vaccinated in time. The effective reproduction number *R_υ_* was 0.91. A fraction of 91.8% of transmissions took place in the casual contact ring. The mean number of infected persons during the epidemic was 101 (range 2–663), excluding the infected contacts vaccinated in time and 340 (range 2–2,175 persons) including those contacts. The mean time to extinction was 229 days (range 32 to >900 days). The time to extinction can be very long when *R_υ_* is near 1 because the epidemic can flare up again when a case by chance produces many secondary infections. A similar picture would result if, in addition to vaccination, contacts are monitored. However, if vaccination coverage is 55% in the casual contact ring, the effective reproduction number is 0.96; 93.0% of transmissions are in the casual contact ring.

### Sensitivity Analysis

#### Initial Phase of Epidemic

**Figure 4:**
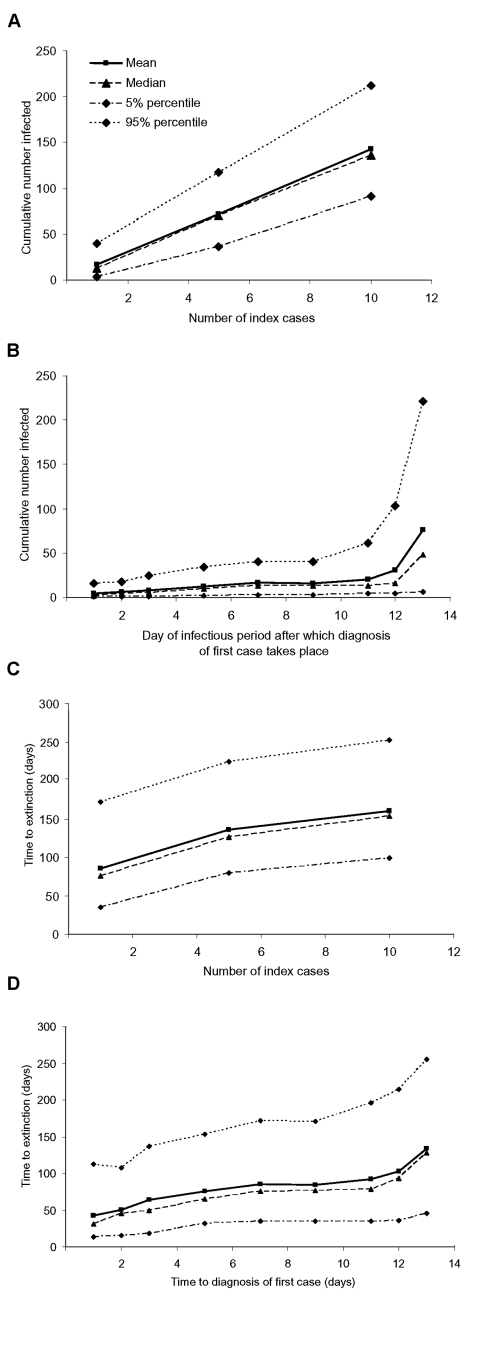
Results for the sensitivity analyses. The total number of infected persons (excluding successfully vaccinated infected contacts) depends on A, the number of index cases starting the epidemic, and B, the day of the infectious period after which the diagnosis of the first case occurs. The time to extinction is shown for C, different numbers of index cases, and D, the day of the infectious period after which the diagnosis of the first case occurs. The quantiles are taken pointwise for 500 simulation runs.

The initial phase of the epidemic (time before discovery of the first case) is determined by the number of index patients that start the epidemic outbreak and by the time it takes to diagnose the first case. We varied those two variables separately while assuming that after diagnosis intervention took place within the parameters defined in the Table, i.e., with an *R_υ_* of 0.67 (Figure 4). While the number of cases increases almost linearly with the number of index cases, the dependency on the time to diagnosis shows the influence of the variable infectiousness during the infectious period. In the beginning, when infectiousness is high, the number of infected persons increases rapidly. Another rise occurs toward the end of the index patient’s infectious period because the second-generation patients become infectious and produce the third generation of infected persons. Once the second generation of infected persons has the opportunity to disseminate the infection further, the range of possible outcomes increases greatly (range 6–221 infected persons). A similar picture emerges for the time needed to extinguish the outbreak. The duration of the outbreak increases when diagnosis is delayed during the first few days of infectiousness, then stays on a stable level, and finally increases again when diagnosis is delayed towards the end of the infectious period (Figure 4d). Diagnosing the first index case before the second generation of infected persons start transmitting the virus is important. If diagnosing the first case at the beginning of its infectious period is possible, the number of cases during the epidemic can be kept at a low level.

### Effectiveness of Response

**Figure 5:**
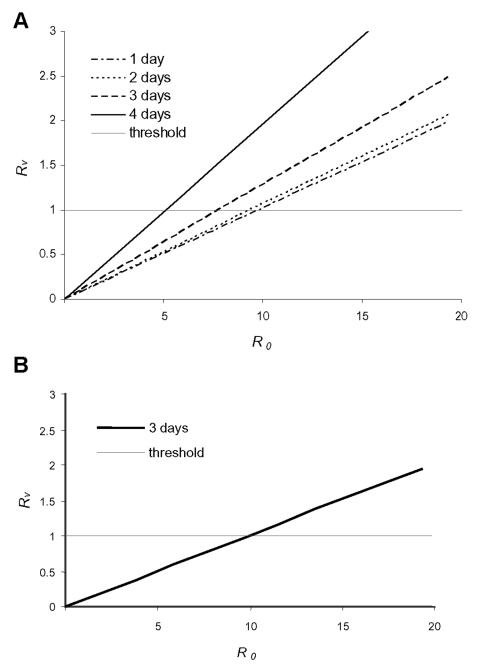
The effective reproduction number *R_υ_*, that determines the success of intervention is shown as a function of the basic reproduction number *R_0_* for a vaccination coverage of 50% in the casual contact ring. In A, contacts are not monitored after vaccination; in B, all identified contacts are isolated and cause not further transmission. The different lines in A are for different assumptions about how long it takes to trace and vaccinate those contacts. In B, it does not make a difference whether it takes 1, 2, or 3 days to find the contacts. If *R_0_* is 5, the intervention will be successful in both cases, if *R_0_* is 10, 50% coverage is no longer sufficient any longer to curb the epidemic.

Among others, the value of *R_0_* determines whether ring vaccination as defined above can contain an epidemic or not. As the value of *R_0_* is uncertain (*2*,*9*,*19*), we studied *R_υ_* as a function of *R_0_* (Figure 5). In Figure 5a, an intervention without monitoring of contacts is considered with various assumptions on how long tracing and vaccinating casual contacts take. In this case, ring vaccination can contain the epidemic if *R_0_* is <7, and contacts can be traced within 3 days. In Figure 5b, monitoring of contacts is added to the intervention. In that instance, the epidemic can be contained up to an R*_0_* of 10. In Figure 5a and b, we assumed that 50% of all casual contacts can be identified and vaccinated or monitored.

**Figure 6:**
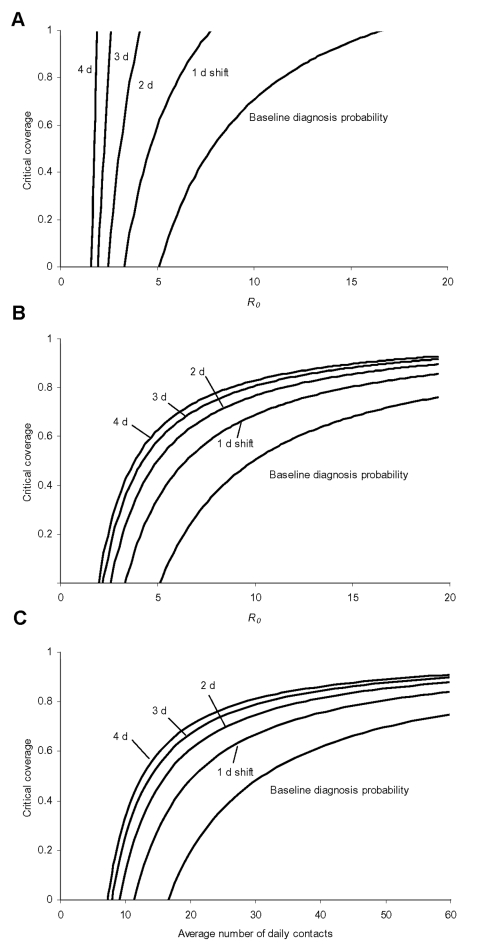
Here the critical vaccination coverage in the casual contact ring is shown as a function of the basic reproduction number *R_0_* for different assumptions about the time it takes to diagnose infectious persons. A, for the baseline assumption, that diagnosis is very quick after the beginning of the infectious period, a low coverage is sufficient if *R_0_* is 5, but for *R_0_* around 10 the coverage has to be at least 70% for the intervention to be successful. If the probability of being diagnosed shifts to later days of the infectious period, the situation quickly gets out of control and vaccination can no longer curb the epidemic. In B, the same is shown with the difference that here we assume that vaccinated contacts are successfully monitored such that they can no longer produce any secondary infections, even if their vaccination was too late to prevent them from becoming infectious. In this case a later diagnosis is not that influential, but nevertheless, if *R_0_* is 10, the vaccination coverage (or the percentage of contacts identified and monitored) must be at least 50% to guarantee success. In C, the critical coverage of the casual contact ring is shown as a function of the average number of contacts per day, again for the situation where vaccination is combined with monitoring of contacts. The average number of contacts was varied by varying the number of daily casual contacts. The effect is the similar to that of varying *R_0_* through the transmission probability per contact as shown in B.

In Figure 6, we show how the critical vaccination coverage needed to control the epidemic depends on *R_0_*, or, more specifically, on the average number of daily contacts (Figure 6c). In addition, we varied the baseline assumption about the time to diagnosis by shifting the probability of being diagnosed by n days towards a later time in the symptomatic period. In Figure 6a, without monitoring of vaccinated contacts, a shift by 1 day increases the coverage needed to contain the epidemic. If diagnosis is delayed by >1 day, the chances of controlling the epidemic diminish greatly. If vaccinated contacts are monitored, the situation improves (Figure 6b), and a high vaccination coverage in the casual contact ring ensures that the epidemic stays under control.

### Time to Extinction Depending on R_υ_

**Figure 7:**
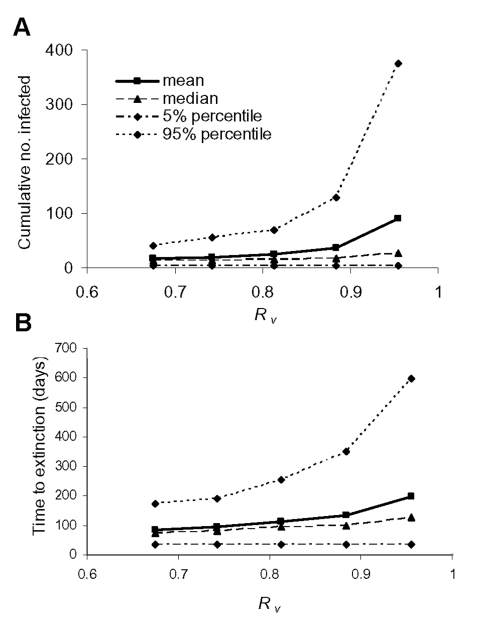
A, the cumulative number of infected persons (excluding successfully vaccinated infected contacts), and B, the time to extinction are shown for various values of the effective reproduction number *_Rυ_*. The quantiles are taken pointwise for 500 simulation runs.

Finally, we looked at how differences in intervention effectiveness influence the duration of the epidemic and the cumulative number of infected persons. The effective reproduction number *R_υ_* was varied by decreasing the vaccination coverage in the casual contact ring stepwise to 0.1. The effective reproduction number increased up to 0.96 (starting from the baseline value of 0.67). Figures 7a and b show how the cumulative number of infected persons and the time to extinction increase with increasing *R_υ_.* The mean time until extinction approximately doubles to almost 200 days, and the range of possible outcomes increases with maximum possible durations of >2 years. The mean number of infected persons increases by a factor of 5, and the range of possible outcomes increases such that epidemics with several hundreds of infected persons are possible. Hence, if *R_υ_* is slightly <1, the epidemic might take a long time to control, and the number of persons who become infected and die might be high.

## Discussion

Our simulation results show that a smallpox epidemic starting from a small number of index cases can be contained by ring vaccination provided the intervention measures are very effective. The time to diagnosis has proven to be an essential and sensitive parameter in determining the intervention effectiveness. The speed of diagnosis is less essential if identified contacts are isolated to prevent them from transmitting further if their vaccination fails. The time window limiting the success of vaccination then loses its importance for determining the effectiveness of intervention. The time to diagnosis of cases and the fraction of contacts found by contact tracing are then the key parameters. Contact tracing would be even more essential if substantial transmission would take place during the prodromal period of infection as is assumed by some authors (*11*,*12*).

Some limitations of our modeling approach should be kept in mind. First, we only consider epidemics that are started by a small number of index cases. The branching process approach does not allow for overlapping rings of contact, but we implicitly include such an effect by varying the effective transmission probability such that the distribution of transmissions over the infectious period agrees with empirical findings (*8*). In other words, the decreasing probability of contacting new susceptible persons during the infectious period is incorporated in the decreasing transmission probability per contact. For larger numbers of index cases, our approach can be viewed as a worst-case scenario. Second, we assume that the population is completely susceptible, i.e., no residual immunity from vaccination in the pre-eradication era exists. This lack of immunity means that if previously vaccinated persons cannot become infectious to others, our results are too pessimistic, whereas if they become infectious with mitigated symptoms, our results might be too optimistic.

In the recent literature, other models, both stochastic and deterministic, of smallpox outbreaks have been introduced to analyze the effects of ring and mass vaccination (*8*,*10*–*14*). On the basis of a low estimate for *R_0_*, Meltzer et al. (*8*) concluded that even quarantine alone can control the epidemic, as can vaccination alone, if the transmission rate is reduced sufficiently. However, the model does not allow analysis of how intervention parameters determine the reduction of the transmission rate. On the other hand, Kaplan et al. (*10*) conclude that with a large number of initial cases, mass vaccination will prevent more deaths than will a vaccination strategy based on contact tracing. Those authors explicitly take into account the limited resources available for tracing and vaccinating contacts, a limitation that is an important factor in large outbreaks. Also, they assume that infectivity is high during the prodromal phase. As in the model by Kaplan et al. (*10*), our model takes into account that the intervals between infection and diagnosis of an index case, and between diagnosis of an index case and tracing of the contact, may exceed the time window in which vaccination has to take place. This window limits the possible effectiveness of contact vaccination—a phenomenon termed “race to trace” by Kaplan et al. While the model by Kaplan et al. is based on differential equations with exponentially distributed sojourn times in different compartments, our model is a stochastic model that is able to deal with more realistic distributions for sojourn times in different disease states. Also, our model can provide estimates for variability in outcomes (Discussion in [*25*]).

In a study by Halloran et al. (*11*), a stochastic model for smallpox outbreaks in small structured communities is described. In some respects the model is similar to ours, namely, that there is a distinction between household contacts and other contacts in the community with differing transmission probabilities. The values of most biologic parameters are choices similar to ours, with the exception of Halloran’s assumption that persons are highly infectious during the prodromal phase. An important difference between the models is the natural limitation of the number of infected persons in any epidemic attributable to the rather small community size in Halloran’s model. The nonlinear effects of saturation play a rather large role in determining the outbreak size, especially for less effective intervention measures. Also, Halloran’s model seems to be too complex to derive an explicit formula for the basic reproduction number *R_0_*, and thus makes sensitivity analysis of the results based on that quantity much more tedious. One of the main differences, however, is the way that vaccination is incorporated into the model does not allow investigation of the effects of those parameters that largely determine the success of intervention, namely the time to diagnosis of new cases and the time needed to trace contacts. In a small closed community as the one described in the model, tracing of contacts is not that difficult, but in modern society with its increasing mobility the tracing of casual contacts can pose a big problem.

The main difference between the modeling approach of Bozette et al. (*12*,*13*) and our model is that in the Bozette’s *R_0_* and *R_υ_* are set to prescribed values, while in our model those numbers can be derived from measurable quantities inherent to the transmission and intervention process. In comparison to the estimate of *R_υ_* of 0.53 in our baseline scenario with vaccination and monitoring of contacts, Bozette et al. assume a much lower value of 0.1. So compared to our results, their results are rather optimistic, but they cannot relate the assumed value of *R_υ_* to *R_0_* or to parameters describing the process of contact tracing and vaccination.

Finally, Eichner (*14*) recently published a modeling study that uses a simulation model to assess the effectiveness of case isolation and contact tracing. Modeling approach and choice of parameter values resemble our approach, but the intervention is modeled in a more phenomenologic way by defining the outcomes of intervention without explicitly including intervention-related parameters into the model. This does not allow for an explicit calculation of the effective reproduction number based on intervention parameters as is possible in our approach.

With respect to preparing for a smallpox outbreak, alertness and ability to diagnose quickly are important. Physicians and nurses need to be educated and the public needs to be more aware. Also, since we know little about the timing and effectiveness of identifying infectious persons and their contacts in case of a bioterror attack, obtaining more empirical information about contact patterns and contact tracing will be helpful. Recently, some useful data about contact patterns have been collected during severe acute respiratory syndrome outbreaks, but a more systematic investigation of contact tracing is advisable. Considering the uncertainties connected to all parameter values, we conclude that any contingency plan for use of ring vaccination must also identify the criteria under which switching to large-scale mass vaccination is justified.

## Supplementary Material

Appendix Formal Model Definition
