# Supplementary material for: Ring Vaccination and Smallpox Control
Source: Emerg Infect Dis. 2004 May;10(5):832–41. doi: 10.3201/eid1005.030419 (PMC3323203; doi:10.3201/eid1005.030419)
Supplement: Appendix — Formal Model Definition [file 03-0419-app-s1.pdf]

Appendix

We used a stochastic model to describe the number of infected persons following the introduction of one or more index cases. The model simulates a discrete time-branching process.

Formal Model Definition

By $E_{t,t}$ we denote the number of persons infected at time t-t who are still latent at time t, $1 \leq t \leq D_E$. By $I_{t,t}$ we denote the number of persons who became symptomatic at time t-t and are not yet in isolation at time t, $1 \leq t \leq D_I$. By $Q_{t,t}$ we denote the number of persons who became symptomatic at time t-t and are in isolation at time t, $1 \leq t \leq D_I$.

S_t denotes a Bernoulli distributed random variable with mean $?_t$, $1 \leq t \leq D_E$, where $?_t$ is the probability to move to the infectious state on day t of the latent period. D_E is the maximum duration of the latent period.

T_t denotes a Bernoulli distributed random variable with mean p_t, $1 \leq t \leq D_I$, where p_t is the transmission probability per contact on day t of the infectious period. D_I is the duration of the infectious period. We assume that p_t can be well described by the functional form $p_\tau = a_1 e^{(-a_2 \tau)}$ with a_1, $a_2 > 0$.

$C_t^{(1)}$ denotes a Poissson distributed random variable with mean $\mu_\tau^{(1)}$, $1 \leq t \leq D_I$ describing the number of contacts per day in the close contact ring.

$C_t^{(2)}$ denotes a random variable with a negative binomial distribution with parameters n_t and q_t, $1 \leq t \leq D_I$ describing the number of contacts per day in the casual contact ring. The mean number of contacts per day in the casual contact ring is $\mu_\tau^{(2)} = n_\tau(1-q_\tau)/q_\tau$ and the standard deviation is $\sigma_\tau = \sqrt{n_\tau(1-q_\tau)}/q_\tau$.

The transition from being undiagnosed infectious to isolation depends on the probability of diagnosis $?_t$ on day t after the start of the infectious period. $?_t$ is a Bernoulli distributed random variable with parameter d_t, t=$1,...,D_I$.

$V_t^{(1)}$ and $V_t^{(2)}$ are Bernoulli distributed random variables with parameters $?_t^{(1)}$ and $?_t^{(1)}$, respectively, with t=$1,..., D_I$. They describe the probability that a contact in ring 1 or 2 , respectively, will be effectively vaccinated within the time window of 4 days after infection. The subscript t refers to day of the infectious period of the index case at the moment of transmission. The vaccination probabilities $?_t^{(i)}$ for i=1,2 depend on the probability that the index case is diagnosed and on the vaccination coverage as follows. The probability $\overline{\delta_{\tau,j}}$ an infectious person who has transmitted to a contact on day t of the infectious period is diagnosed on day t+j can be computed as

$$\overline{\delta_{\tau,j}} = \delta_{\tau+j} \prod_{k=\tau}^{\tau+j-1} (1-\delta_k)$$ for $j=1,..., D_I-t+1$.

The probability for the infected contact to be vaccinated in time depends on the number of days w after infection that vaccination can still be effective, the number of days $r^{(i)}$ that are needed for tracing the contact, and the coverage $c^{(i)}$ (the fraction of contacts in ring i that are effectively immunized) for i=1,2. Then we get

$$U_\tau^{(i)} = \sum_{k=1}^{Min(D_I-\tau+1,\tau+w-1-r^{(i)})} \overline{\delta_{\tau,k}} \quad c^{(i)}$$ for i=1,2.

To describe the probability of diagnosis per day of the infectious period we use the functional form

$\delta_\tau = 1 - e^{-b_1(\tau-b_2)}$ for t > b_2, and $\delta_\tau = 0$ for t < b_2

with $b_1 > 0$, and an integer b_2 between 0 and D_I. In the simulations different values for those parameters are chosen for the first index patient, who starts the epidemic, and later cases assuming that it takes longer to diagnose the first case as there is not yet that much alertness of the public health system. Also, the time needed to find contacts and the vaccination coverage may vary between the first index case and later cases.

The transitions through the different stages in time is described by the system of difference equations.

$$E_{t+1,\tau+1} = E_{t,\tau} - \sum_{k=1}^{E_{t,\tau}} S_{k,\tau}, \qquad \tau = 1,\ldots,D_E$$

$$I_{t+1,\tau+1} = I_{t,\tau} - \sum_{k=1}^{I_{t,\tau}} \Delta_{k,\tau}, \qquad \tau = 1,\ldots,D_I$$

$$Q_{t+1,\tau+1} = Q_{t,\tau} + \sum_{k=1}^{I_{t,\tau}} \Delta_{k,\tau}, \qquad \tau = 1,\ldots,D_I$$

The inflow of new latent and symptomatically infected persons is given by

$$E_{t+1,1} = \sum_{\tau=1}^{D_I} \sum_{k=1}^{I_{t,\tau}} \left(\sum_{n=1}^{C_\tau^{(1)}} T_{k,n,\tau}(1 - V_{k,n,\tau}^{(1)}) + \sum_{n=1}^{C_\tau^{(2)}} T_{k,n,\tau}(1 - V_{k,n,\tau}^{(2)}) \right)$$

$$I_{t+1,1} = \sum_{\tau=1}^{D_E} \sum_{k=1}^{E_{t,\tau}} S_{k,\tau}$$

The initial conditions for the case that the epidemic is started by one infected index case entering the population at the beginning of his latent period are given by

$E_{0,1} = 1,$

E $= 0$ for $t = 2,\ldots, D$,

$_{0,t}$ $_E$

$I_{0,t} = 0$ for $t = 1,\ldots, D_I$, and

$Q_{0,t} = 0$ for $t = 1,\ldots, D_I$.

At the end of the infectious period an infected patient either recovers and becomes immune or dies. Death occurs in a fraction *f* of all cases, i.e., *f* denotes the case fatality. This implies that the number of deaths M_{t+1} at time $t+1$ is given by

$$M_{t+1} = \sum_{k=1}^{I_{t,D_I}} Z_k ,$$

where Z is a Bernoulli distributed random variable with parameter *f* , the case-fatality rate. The cumulative mortality from the start of the epidemic up to time t is given by $\overline{M}_t = \sum_{n=1}^{t} M_n$.

The model was implemented and run in Mathematica 4.2.
